# Anxiety and Somatic Symptoms in Children and Adolescents Diagnosed with Attention-Deficit Hyperactivity Disorder

**DOI:** 10.1155/2023/5523312

**Published:** 2023-08-07

**Authors:** Esra Okyar, Leyla Bozatlı, Işık Görker, Serap Okyar

**Affiliations:** ^1^Department of Child and Adolescent Psychiatry, Sakarya Training and Research Hospital, Sakarya, Turkey; ^2^Faculty of Medicine, Department of Child and Adolescent Psychiatry, Trakya University, Edirne, Turkey; ^3^Faculty of Medicine, Department of Public Health, Sakarya University, Sakarya, Turkey

## Abstract

**Background:**

Attention-deficit hyperactivity disorder (ADHD) is a neurodevelopmental condition typified by inattention, hyperactivity, and impulsivity. Comorbid psychiatric disorders are common among children and adolescents with ADHD. In this study, it was aimed to examine anxiety and somatic symptoms in children and adolescents with ADHD and the effect of methylphenidate treatment on these symptoms.

**Method:**

Three groups were formed, consisting of 37 children and adolescents diagnosed with ADHD and received methylphenidate treatment, 37 newly diagnosed, treatment-naive children and adolescents with ADHD diagnosis, and 37 children and adolescents without the diagnosis of ADHD. The symptoms of ADHD in children were examined by using the DSM-IV-based child and adolescent behavior disorders screening and rating scale, the symptoms of anxiety were examined by using the screen for child anxiety-related disorders (SCARED), and somatic symptoms were examined by using the DSM-5 level 2 somatic symptom scale.

**Results:**

In the newly diagnosed, treatment-naive with ADHD group, anxiety and somatic symptoms were found to be significantly higher compared to the ADHD group with methylphenidate treatment and the non-ADHD group. It was shown that the symptoms of panic disorder, generalized anxiety, and social phobia were observed more in the newly diagnosed, treatment-naive with ADHD group compared to the treatment group with ADHD.

**Conclusions:**

It was determined that children and adolescents diagnosed with ADHD had more anxiety and somatic symptoms. Anxiety and somatic symptoms increased as the severity of ADHD symptoms increased. Anxiety and somatic symptoms were lower than in ADHD children receiving methylphenidate treatment. Clinicians should keep in mind to evaluate anxiety and somatic symptoms in children and adolescents with ADHD before the treatment.

## 1. Introduction

Attention-deficit hyperactivity disorder (ADHD) is identified as a neurodevelopmental disorder typified by symptoms of inattentiveness, hyperactivity, and impulsiveness. These symptoms negatively affect the social, school, and work-related activities [1]. The prevalence of ADHD is between 3% and 7% [2]. As a result of a systematic review, the worldwide prevalence of ADHD was found to be 5.29% [3]. Boys are more likely to receive an ADHD diagnosis than girls, the male-to-female ratio varies between 1 : 1 and 3 : 1 in the population sample and is 9 : 1 in clinical studies [4].

Comorbid psychiatric disorders accompany 40-80% of children with ADHD [5]. Jehnsen and Steinhausen demonstrated that 52% of patients diagnosed with ADHD had at least one psychiatric comorbidity, and 26.2% of them had two or more comorbid conditions. While neuropsychiatric disorders are more common in boys, internalizing disorders are more common in girls [6]. The most common comorbid psychiatric conditions accompanied in children with ADHD are oppositional defiant disorder (ODD), conduct disorder (CD), autism spectrum disorder, depression, anxiety disorder, bipolar disorder, tic disorder, and obsessive-compulsive disorder [5–8]. More internalizing and externalizing disorders are observed in the combined type ADHD [9]. In patients where ADHD with comorbid disorders, more impairment of mental health, decreased quality of life, and psychosocial impairment are observed [7–10].

The incidence of anxiety disorder is 6.5% in children and adolescents [11]. The coexistence of anxiety disorder and ADHD varies between 10-40% in the clinical sample [5]. The prevalence of anxiety disorder in cases with ADHD increases after puberty. This increase is higher especially in girls [12]. ADHD increases the risk for poor academic performance and poor social, emotional, and adaptive functioning, which may take part in the start of the anxiety symptoms [13]. Individuals with ADHD and anxiety disorder are predisposed to have more severe and early onset of anxiety symptoms and accompanying other psychiatric disorders [14]. ADHD and anxiety disorder comorbidity may change the clinical presentation, prognosis, and treatment of ADHD. To choose the most appropriate therapeutic treatment in cases with ADHD and anxiety disorder comorbidity may be difficult for clinicians [15]. The treatment of a disorder may complicate or disturb the treatment of the other disorder [16]. In these cases, better response is obtained when drug treatments and therapeutic treatments are used together [5, 7, 15].

Somatic symptoms are common in children and adolescents [17]. It was demonstrated that 20% and 30% of adolescent girls had morning fatigue, stomachache, headache, and backache more than once a week [18]. Children and adolescents with ADHD diagnosis have somatic complaints; nevertheless, somatic complaints can be ignored by the clinicians [19, 20]. In a study conducted with male adolescents aged 12-16 years, it was reported that adolescents with hyperactivity symptoms had more somatic complaints [20].

Psychostimulants are the first choice in the treatment of ADHD [21]. Studies examining the effects of methylphenidate on anxiety have different results. Some studies have found no difference with methylphenidate treatment between children with ADHD and children with ADHD+anxiety [22]. In a study, it has been observed that methylphenidate treatment reduces both anxiety and ADHD symptoms [23]. In another study, it has been shown that as the level of anxiety increases in children with ADHD, the methylphenidate response decreases [24].

Due to children and adolescents with AHDH diagnosis having anxiety and somatic symptoms, and to our knowledge, there are limited studies on this issue, it was aimed to examine the frequency and dispartion of anxiety and somatic symptoms in children and adolescents with ADHD. It was also aimed to investigate the methylphenidate treatment effect on anxiety and somatic symptoms in children and adolescents with ADHD.

## 2. Methods

### 2.1. Participants

A total of 111 children and adolescents, including 37 newly diagnosed, treatment-naive children and adolescents with ADHD between the ages of 6 and 15, 37 children and adolescents diagnosed with ADHD receiving only methylphenidate (extended-release or immediate-release formulations) treatment for at least six months, and 37 children and adolescents without the diagnosis of ADHD as the control group, participated in the current study. Newly diagnosed, treatment-naive children with ADHD did not use any psychotropic medication. A psychiatric interview based on DSM-5 was held by a child and adolescent psychiatrist with all participants. All participants' parents filled out the DSM-IV-based child and adolescent behavior disorders screening and rating scale in order to assist the diagnosis of ADHD. The sociodemographic data form was given to parents to obtain the sociodemographic data. The parents filled out the screen for child anxiety and related disorder- (SCARED-) parent form to examine the symptoms of anxiety and the DSM-5 level 2 somatic symptom scale-parent form to examine somatic symptoms. Those with a score of 25 and above in the SCARED were excluded. Somatic symptoms were also evaluated by clinician in all participants. The patients who had an IQ score of less than 80, known neurological, genetic, and chronic medical diseases, and used medication related to it were not included. Also, having mental retardation, bipolar disorder, and psychosis were determined as exclusion criteria in all groups. The study was approved by the Trakya University Local Ethics Committee (TÜTF-BAEK 2019/400). All participants were informed about the current study, and their written consent was obtained.

### 2.2. Measurements

#### 2.2.1. DSM-IV-Based Child and Adolescent Behavior Disorders Screening and Rating Scale

It is a 41-item scale developed by converting the DSM-IV diagnostic criteria into a question form. The scale has 9 items examining attention deficit, 6 items examining hyperactivity, 3 items examining impulsivity, 8 items examining ODD, and 15 items examining CD. To confirm the diagnosis of ADHD, at least 6 of 9 items questioning attention deficit should be rated as 2 or 3, or at least 6 of 9 items questioning hyperactivity and impulsivity should be rated as 2 or 3. The Turkish validity and reliability study of this scale has been carried out by Ercan et al. [25].

#### 2.2.2. Screen for Child Anxiety and Related Disorder- (SCARED-) Parent Form

It consists of 41 items and 5 subscales (panic disorder/somatic disorder, generalized anxiety, separation anxiety, school phobia, and social phobia). It is accepted that a score of 25 and above in the SCARED is a warning for anxiety disorder. Its Turkish validity and reliability study has been carried out by Çakmakçı et al. [26].

#### 2.2.3. DSM-5 Level 2 Somatic Symptom Scale-Parent Form

The parent form for children aged 6-17 is a Likert-type scale that evaluates the somatic symptoms in the last seven days. The total score is between 0 and 26, and higher scores indicate more severe somatic symptoms. Its Turkish validity and reliability study has been carried out by Sapmaz et al. [27].

### 2.3. Statistics

Mean, median, minimum and maximum values, standard deviation, and percentage were used in descriptive statistics. The Mann–Whitney *U* and Kruskal-Wallis tests were used for the analysis of quantitative independent data. For analyzing qualitative independent data, the chi-square test was used. The relationship between the variables was analyzed using the Spearman correlation analysis. IBM SPSS 27.0 was used. The limit of significance was chosen as *p* < 0.05 for all statistics.

## 3. Results

### 3.1. Sociodemographic Results

The mean age was 9.1 ± 1.7 in the newly diagnosed, treatment-naive with ADHD group, 9.3 ± 2.1 in the treatment group for ADHD, and 8.8 ± 1.9 in the non-ADHD group. The groups are similar in terms of age and gender (*p* > 0.05). Maternal educational level was lower in the newly diagnosed, treatment-naive with ADHD group (*p* > 0.05). There was no significant difference between the groups in terms of paternal educational level (*p* > 0.05). The distribution of the income levels of the families was similar in all groups (*p* > 0.05) (Table 1).

### 3.2. Scores of the DSM-IV-Based Child and Adolescent Behavior Disorders Screening and Rating Scale

The scores of attention deficit, hyperactivity-impulsivity, ODD, and CD were significantly higher in the newly diagnosed, treatment-naive with ADHD group and treatment group for ADHD compared to the non-ADHD group (*p* < 0.05). The total score was significantly higher in the newly diagnosed, treatment-naive with ADHD group compared to the treatment group with ADHD (*p* < 0.05) (Table 2).

### 3.3. Scores of the Screen for Child Anxiety and Related Disorders

The total score of SCARED was found to be significantly higher in the newly diagnosed, treatment-naïve with ADHD group compared to the treatment group with ADHD and the non-ADHD group (*p* < 0.05). When subscale scores were compared, the scores for panic/somatic symptoms, generalized anxiety, and school phobia were demonstrated to be higher in the newly diagnosed, treatment-naive with ADHD group compared to the treatment group with ADHD (*p* < 0.05). No significant difference was indicated in the panic/somatic symptoms, separation anxiety, generalized anxiety, and school phobia subscale scores between the treatment group with ADHD and the non-ADHD group (*p* > 0.05) (Table 2).

The total score of SCARED is found to be significantly higher in boys than girls in the newly diagnosed, treatment-naïve with ADHD group. No significant difference was found between girls and boys in the total score of SCARED in the treatment group with ADHD and the non-ADHD group.

### 3.4. Scores of the DSM-5 Level 2 Somatic Symptoms Scale

The total score was demonstrated to be 3.09 ± 2.90 in the newly diagnosed, treatment-naïve with ADHD group, 2.06 ± 2.89 in the ADHD group with treatment, and 1.37 ± 1.91 in the non-ADHD group. The total score was found to be significantly higher in the newly diagnosed, treatment-naive with ADHD group (*p* < 0.05). No significant difference was indicated between the ADHD group with treatment and the non-ADHD group (*p* > 0.05) (Table 2).

No significant difference was found between girls and boys in the total score of the DSM-5 level 2 somatic symptoms scale in all groups.

### 3.5. Analysis of the Relationship between Somatic Symptoms and ADHD and Anxiety Symptoms

A significant correlation was shown between somatic symptoms scale total scores and DSM-IV-based child and adolescent behavior disorders screening and rating scale total score (*p* = 0.002). A significant correlation was found between somatic symptom scale total scores and the SCARED total score (*p* = 0.001) (Table 3).

## 4. Discussion

It was observed that the children and adolescents with ADHD had more anxiety and somatic symptoms compared to the non-ADHD children and adolescents in this study which is compatible to the literature. Both anxiety and somatic symptoms were significantly higher in the newly diagnosed, treatment-naive with ADHD group compared to the children and adolescents diagnosed with ADHD receiving methylphenidate treatment. It was observed that anxiety and somatic symptoms increased as the severity of ADHD symptoms increased. In a study conducted with 102 children with ADHD, it was shown that while externalizing symptoms were mostly observed in hyperactive-impulsive type ADHD, anxiety, depression, delinquent behavior, and internalizing symptoms were mostly observed in the combined type. Somatic complaints were more common in boys [28].

The total score of SCARED was significantly higher in the newly diagnosed, treatment-naive with ADHD group compared to the treatment group with ADHD and the non-ADHD group in this study. The symptoms of generalized anxiety, panic disorder, social phobia, separation anxiety, and school phobia were shown to be higher in the newly diagnosed, treatment-naive with ADHD group compared to the non-ADHD group. It was shown that the symptoms of panic disorder, generalized anxiety, and social phobia were observed more in the newly diagnosed, drug-naive ADHD group compared to the ADHD group with treatment. The ADHD group with treatment had more social phobia symptoms compared to the non-ADHD group. Souza et al. demonstrated that 23.5% of the children with ADHD were accompanied by anxiety disorders, and the generalized anxiety disorder (GAD) is the most common accompanying anxiety disorder by 12.8%, followed by social phobia by 3.84%, and separation anxiety by 3.8% in their study [29]. In another research study, it was revealed that 20% of children with anxiety disorder were accompanied by comorbid ADHD [30]. More severe anxiety symptoms, earlier onset, and more frequent other comorbid disorders and substance use are observed in individuals with comorbid ADHD accompanied by anxiety disorder compared to the individuals only diagnosed with ADHD [31]. Anxiety symptoms are found to be significantly higher in boys than girls in the newly diagnosed, treatment-naïve with ADHD group in our study. Although internalization disorders are more common in girls, in a study, it was reported that social anxiety and separation/panic symptoms were more frequent in adolescent males than in adolescent girls [32].

It was shown that anxiety symptoms increased as the severity of ADHD symptoms increased in our study. Taurines et al. showed that the coexistence of ADHD diagnosis and anxiety disorders is associated with more attention problems, school phobia, mood disorder, and lower social ability compared to anxiety alone or ADHD alone [7]. Most of ADHD and comorbid disorders have a heterogeneous etiology including genetic and environmental risk factors [7, 15]. It is recommended that there be a distinct etiology for anxiety and depression that develop in the presence of ADHD [33]. Whether the presence of anxiety disorder is the consequence of ADHD symptoms should be investigated [15]. Having ADHD is associated with low academic performance and increased risk of poor social and emotional functionality, which contributes to the emergence of anxiety symptoms [13]. In preadolescents with combined-type ADHD symptoms and anxiety and depressive symptoms living under chronic stress, the persistence of ADHD and exacerbation of anxiety disorders and depression can be observed in the future [33]. More social anxiety, separation/panic, and physical symptoms were observed in adolescents with ADHD. The relationship between ADHD symptoms and social anxiety symptoms was found to be more significant in younger adolescents than in older adolescents [32]. In a study conducted with children and adolescents with ADHD aged 6-18 years, age was found to be significantly correlated with somatic complaints and internalization problems. Untreated adolescents with ADHD had more internalization problems than untreated children with ADHD [34]. Another study indicated that ADHD symptoms predicted later anxiety symptoms, but anxiety symptoms did not predict later ADHD symptoms [35]. The risk of anxiety disorder is also higher in adults with ADHD [14].

In the current study, somatic complaints were observed to be significantly higher in the newly diagnosed, treatment-naïve children and adolescents with ADHD. Somatic complaints were observed to be less in the ADHD treatment group compared to the newly diagnosed, treatment-naive with ADHD group in the current study. Ahmann et al. demonstrated that irritability, staring, daydreaming, nail-biting, and anxiety were reduced by short-acting methylphenidate treatment. In the same study, appetite disturbance, stomachache, insomnia, headache, and dizziness increased with methylphenidate [36]. Barkley et al. demonstrated that decreased appetite, insomnia, headaches, and stomachaches increased significantly in ADHD children and adolescents with methylphenidate treatment by parent ratings compared to the placebo group. Meanwhile, teacher ratings showed little change in children under methylphenidate treatment except on ratings of anxiety, staring, and sadness, but the placebo group had these side effects too [37]. Shih et al. found that methylphenidate and atomoxetine were effective in improving emotional and behavioral problems in adolescents with ADHD after 24 weeks of treatment, but there were more improvement in somatic complaints in the methylphenidate group [38]. Also, another study showed that methylphenidate treatment improved somatic symptoms in adolescents with ADHD. Kim et al. found that methylphenidate increased brain functional connectivity within the anterior and posterior default mode network, and it was associated with improvement of somatic symptoms [21]. Rapport and Moffitt indicated that somatic complaints should be evaluated separately before the initiation of psychostimulant treatment [39]. The results of the present study indicate that the clinicians should consider evaluating somatic symptoms before starting ADHD treatment and during the treatment process, since somatic complaints are observed more in the newly diagnosed, drug-naive ADHD group compared to the ADHD group with methylphenidate treatment.

There is no significant difference between girls and boys in terms of somatic symptoms in this study. In a study, it was reported that generalized anxiety, phobia, and being a girl were risk factors for somatic symptoms in children with ADHD, and children with ADHD and somatic symptoms had more anxiety symptoms [19]. Also, children with ADHD and anxiety disorder had more physical symptoms than children with only ADHD [40]. The presence of anxiety disorder is a factor that determines the response to ADHD treatment. It is also considered as a side effect of the methylphenidate treatment [41].

## 5. Conclusions

The small sampling is the first limitation of this study for affecting the generability of the results. More studies conducted with larger sampling are needed. The data were obtained only from the parents. The data could be obtained from children and their teachers to increase the data diversity, so this is the second limitation. Making comparisons between three groups is the strength of the study. In conclusion, it was shown that children and adolescents with ADHD had more anxiety and somatic symptoms than children and adolescents without ADHD, and children and adolescents with newly diagnosed, treatment-naive with ADHD had more anxiety and somatic symptoms in this study. Less anxiety and somatic symptoms in children with ADHD who receive methylphenidate treatment may show the protective effect of ADHD treatment from comorbid conditions. Although it is known that somatic side effects are observed with psychostimulant treatment, the clinician should keep in mind that somatic symptoms may also be present before the initiation of the treatment.

Based on the findings of current research, clinicians should evaluate and consider anxiety and somatic symptoms in children and adolescents with ADHD while planning and monitoring methylphenidate treatment. Future studies with larger sampling are needed to investigate anxiety and somatic symptoms in children and adolescents with ADHD and the effect of methylphenidate treatment on these symptoms due to mentioned limitations of this study.

## Figures and Tables

**Table 1:** Comparison of sociodemographic data between the groups.

	Newly diagnosed, treatment-naïve with ADHD		ADHD receiving treatment		Control (non-ADHD)		*p*
	(*n* = 37)		(*n* = 37)		(*n* = 37)		
	Number	Percentage	Number	Percentage	Number	Percentage	
Gender							0.581
Girl	11	29.7	10	27.0	14	37.8	
Boy	26	70.3	27	73.0	23	62.2	
Maternal educational level							0.034^∗^
Uneducated	1	2.7	0	0.0	1	2.7	
Primary school	17	45.9	10	27.0	8	21.6	
Secondary school	7	18.9	4	10.8	5	13.5	
High school	6	16.2	17	45.9	9	24.3	
University	6	16.2	6	16.2	14	37.8	
Paternal educational level							0.891
Uneducated	0	0.0	1	2.7	1	2.7	
Primary school	13	35.1	9	24.3	14	37.8	
Secondary school	1	2.7	3	8.1	0	0.0	
High school	17	45.9	16	43.2	11	29.7	
University	6	16.2	8	21.6	11	29.7	
Monthly income							0.613
<2500 Turkish liras	4	10.8	5	13.5	5	13.5	
2500-3500 Turkish liras	18	48.6	13	35.1	12	32.4	
3500-5000 Turkish liras	5	13.5	11	29.7	11	29.7	
>5000 Turkish liras	10	27.0	8	21.6	9	24.3	

^∗^ *p* < 0.05, chi-square test. ADHD: attention-deficit hyperactivity disorder.

**Table 2:** Comparison of the scale scores of the newly diagnosed, treatment-naive ADHD group, ADHD group with treatment, and the control group.

	Newly diagnosed, treatment-naive ADHD		ADHD with treatment		Control (non-ADHD)		*p*
	Mean ± S.D.	Median	Mean ± S.D.	Median	Mean ± S.D.	Median	
DSM-IV-based child and adolescent behavior disorders screening and rating scale							
Attention deficit	19.59 ± 4.35	19.0	13.49 ± 7.33	14.0	3.41 ± 3.63	2.0	0.001^∗^
Hyperactivity	13.81 ± 6.83	14.0	11.54 ± 6.74	10.0	3.22 ± 4.25	1.0	0.001^∗^
Oppositional defiant disorder	12.43 ± 5.47	13.0	8.00 ± 6.85	7.0	3.89 ± 4.27	3.0	0.001^∗^
Conduct disorder	2.49 ± 3.23	1.0	1.59 ± 3.07	0.0	0.22 ± 0.53	0.0	0.001^∗^
Total	48.32 ± 14.62	46.0	34.62 ± 19.53	31.0	10.73 ± 9.61	9.0	0.001^∗^
SCARED							
Panic/somatic symptom	3.16 ± 3.46	2.00	1.78 ± 2.69	1.00	1.59 ± 2.30	1.0	0.038^∗^
Separation anxiety	4.81 ± 3.20	4.00	3.68 ± 2.42	4.00	2.68 ± 2.61	2.0	0.007^∗^
Generalized anxiety	5.24 ± 6.61	5.00	3.35 ± 3.44	2.00	2.70 ± 3.20	2.0	0.001^∗^
Social phobia	6.11 ± 3.36	6.00	4.86 ± 3.83	5.00	3.05 ± 3.09	2.0	0.001^∗^
School phobia	1.97 ± 2.15	1.00	0.81 ± 1.48	0.00	0.51 ± 0.76	0.0	0.001^∗^
Total	21.30 ± 11.44	20.00	14.49 ± 9.96	13.00	10.62 ± 9.78	9.0	0.001^∗^
DSM-5 level 2 somatic symptom scale							
Total	3.09 ± 2.90	2.31	2.06 ± 2.89	1.15	1.37 ± 1.91	1.2	0.012^∗^

^∗^ *p* < 0.05, Kruskal-Wallis test. S.D.: standard deviation; SCARED: screen for child anxiety and related disorders.

**Table 3:** Relationship between somatic symptoms and ADHD and anxiety symptoms.

	SCARED		DSM-IV-based child and adolescent behavior disorders screening and rating scale	
	*r*	*p*	*r*	*p*
DSM-5 level 2 somatic symptom scale	0.376	0.001^∗^	0.287	0.002^∗^
SCARED			0.397	0.001^∗^

^∗^ *p* < 0.05, Spearman correlation analysis. SCARED: screen for child anxiety and related disorders.

## Data Availability

The datasets used and/or analyzed during the current study are available from the corresponding author on reasonable request.
